# Histotripsy for Renal Tumours: A narrative review of preclinical and early clinical evidence

**DOI:** 10.1177/17562872261485822

**Published:** 2026-10-07

**Authors:** Anirudh Singh Bhati, Ankur Patel, Ru Yu Tan, Valerie Huei Li Gan, Apoorva Gogna

**Affiliations:** 1 School of Clinical Medicine, St Vincent’s Healthcare Clinical Campus, Faculty of Medicine and Health, UNSW Sydney, Sydney, NSW, Australia; 2 Department of Vascular and Interventional Radiology, 37581 Singapore General Hospital, Singapore, Singapore; 3 Duke-NUS Graduate Medical School, Singapore, Singapore; 4 Department of Renal Medicine, 37581 Singapore General Hospital, Singapore, Singapore; 5 Department of Urology, 37581 Singapore General Hospital, Singapore, Singapore; 6 SingHealth Duke-NUS Transplant Centre, Singapore, Singapore

**Keywords:** histotripsy, renal cell carcinoma, RCC, focused ultrasound, non-thermal ablation, acoustic cavitation, renal mass, nephron-sparing, transcutaneous ablation, HOPE4KIDNEY, CAIN

## Abstract

Partial nephrectomy is the reference standard for localised renal cell carcinoma (RCC), but a subset of patients are unsuitable. Percutaneous ablation provides local control, yet is limited by access, tumour anatomy, and heat-sink effects. Histotripsy is a non-invasive, non-thermal focused ultrasound technique and may expand options for focal treatment of RCC. We undertook a narrative review informed by a structured literature search to synthesise in vivo and early clinical evidence for renal histotripsy, focusing on mechanism, feasibility, safety, and implementation barriers. PubMed, Embase (Ovid), and Scopus were searched (2004–December 2025) with trial registry and reference screening. Eligible in vivo animal or human studies of cavitation-cloud or boiling histotripsy targeting renal tumours or related renal structures were summarised descriptively. The search identified 225 records; 17 studies were included. Foundational normal-kidney rabbit and porcine work characterised cavitation thresholds, lesion evolution, and tolerance of adjacent structures. Translational tumour-bearing models (VX2 and Eker RCC) then demonstrated effective tumour fractionation with relative preservation of larger vessels and the renal pelvis, with several studies reporting treatment-associated inflammatory and adaptive immune changes. Large-animal studies supported transcutaneous delivery, feasibility under anticoagulation, and preservation of renal function. Early clinical studies (CAIN) in selected RCC tumours ≤3 cm reported technical success and minor, self-limiting adverse events. Challenges included acoustic-window dependence, respiratory motion mitigation, general anaesthesia, and treatment times up to 80 minutes. A subsequent multicentre study (HOPE4KIDNEY) has completed recruitment and is anticipated to clarify clinical success, safety, and longer-term efficacy. If favourable, it could represent an important step towards broader renal indications. In conclusion, renal histotripsy appears technically feasible with encouraging short-term safety in selected RCC tumours, but evidence remains heterogeneous and early-phase. Future studies should optimise delivery and extend evaluation to more complex tumours, with follow-up that better defines durability, renal functional preservation, and patient experience.

## Introduction

Renal cell carcinoma (RCC) is the seventh most common form of neoplasm in the developed world and has the highest mortality rate of urological cancers.^1^ In 2022, an estimated 434,840 new kidney cancer cases and 155,953 deaths occurred worldwide, with an age-standardised incidence rate of 4.4 per 100,000 and higher rates in high-income regions.^2^ Modern presentations of RCC are often clinically silent, with most cases identified as small, localised renal masses detected incidentally through widespread cross-sectional imaging.^3^

For early-stage disease, contemporary management is individualised. Partial nephrectomy (PN) remains the standard of care for clinical T1 RCC, and selected T2 tumours.^4^ When feasible, PN offers preservation of renal function with excellent oncological control, with 5-year survival rates approaching 98%.^5^ However, a significant proportion of patients are unsuitable for PN. Some are poor candidates for surgery altogether due to elevated perioperative risk associated with advanced age, multimorbidity, or impaired renal function.^6^ Others may face complex or unfavourable tumour anatomy requiring radical resection or even nephrectomy.^7^ Active surveillance is also an established option for selected patients with cT1a renal tumours, in whom intervention may be deferred, particularly where the anticipated risks of treatment or competing mortality outweigh the oncological benefit of immediate intervention.^4,8^

For patients who require active treatment but are unsuitable for, or decline, surgery, minimally invasive focal treatments such as radiofrequency ablation (RFA), microwave ablation (MWA), cryoablation, and stereotactic body radiotherapy (SBRT) have emerged as alternatives to surgery for small renal masses.^9^ Across modalities, focal tumour ablation demonstrates high efficacy, with 5-year local control rates of up to 95% and cancer-specific survival between 95% and 100%.^10^ Accordingly, these techniques have been incorporated into clinical guidelines as evidence-based options for the management of localised RCC. Both the 2021 American Urological Association guidelines and the 2025 European Association of Urology update recognise thermal ablation as an option for selected patients with small renal tumours (typically ≤3–4 cm). The 2025 EAU guideline additionally recognises SBRT for selected patients with cT1 disease who require treatment but are unfit for surgery.^4,8,11^

Thermal ablation techniques, including RFA and MWA, achieve tumour destruction through heat-induced coagulative necrosis.^12^ RFA generates cytotoxic temperatures via high-frequency alternating electrical currents, whereas MWA uses electromagnetic energy to rapidly increase intra-tumoral temperatures.^13^ In contrast, cryoablation induces tumour cell death through repeated cycles of rapid freezing and thawing, leading to intracellular ice crystal formation, membrane disruption, and irreversible cellular injury, with emerging evidence suggesting a potential immunomodulatory effect.^14,15^ However, the physical principles underlying these techniques limit their use across different tumour sizes, shapes, and anatomical locations.^16^ Thermal ablation techniques are susceptible to the heat-sink effect in tumours proximal to large vessels, where adjacent blood flow can dissipate thermal energy and result in incomplete peripheral ablation and residual disease.^17,18^ Cryoablation similarly faces challenges in achieving uniform lethal temperatures that can penetrate larger tumours, limiting its effectiveness.^19^ Furthermore, these methods provide limited real-time feedback during procedures and treatment is limited to tumours that are accessible percutaneously. SBRT is a promising non-invasive ablative therapy for inoperable RCC, using multiple focused radiation doses with recent series reporting 5-year local failure rates of only 5.5%.^20^ Despite these advances, there remains an unmet need for fully non-invasive treatments that avoid ionising radiation while delivering precise and effective tumour ablation.

Histotripsy has emerged as a promising modality to fill this gap, offering a non-invasive, non-ionising, non-thermal, ultrasound-based ablation technique guided by real-time imaging.^21^ By generating sharply defined zones of tissue fractionation while largely sparing vessels and the collecting system, it may be particularly suited to irregular lesions and tumours at the renal hilum or adjacent to other critical structures.^22^ Beyond local control, preclinical work also suggests that histotripsy can enhance the efficacy of immunotherapy and chemotherapy, and trigger broader systemic anti-tumour responses.^23^

This narrative review provides an overview of histotripsy as an emerging focal therapy for renal tumours and its possible role in reshaping nephron-sparing care. It aims to consolidate key preclinical in vivo research and ongoing early-stage clinical trials to illustrate the current evidence for efficacy and safety, while also outlining the limitations and uncertainties.

## Methods

### Design

A narrative review informed by a structured literature search was conducted to provide an overview of histotripsy for renal tissue and RCC. The aim was to summarise key translational studies rather than to perform a systematic or scoping review; accordingly, duplicate independent screening, formal risk-of-bias assessment, and PRISMA reporting were not applied.

### Search strategy

A comprehensive search was performed in PubMed, Embase (via Ovid) and Scopus for studies published from 2004 to December 2025. A combination of terms such as ‘histotripsy’ or ‘boiling histotripsy’ and ‘kidney’ or ‘renal’ or ‘renal cell carcinoma’ was adopted for each database. A full search strategy is available in the appendix. We also searched ClinicalTrials.gov and the WHO International Clinical Trials Registry Platform for ongoing or completed renal histotripsy trials, and screened reference lists of relevant histotripsy reviews and primary articles.

Eligible reports described in vivo application of histotripsy or boiling histotripsy to (i) primary renal tumours, (ii) normal renal parenchyma, or (iii) the proximal ureter or renal pelvis, in animals or humans. Ex vivo and purely phantom work, and reports of generic thermal high-intensity focused ultrasound without a clearly mechanical histotripsy regimen, were not included.

### Study selection

Title, abstract and full-text screening for relevance was performed by one primary reviewer. Three senior reviewers verified the included evidence set and cross-checked exclusions. This process was not equivalent to duplicate independent screening.

Peer-reviewed articles were prioritised and formed the primary evidence base. Trial protocols and registry records were used to describe study design, eligibility, and recruitment status. Two conference abstracts were retained only where they provided otherwise unavailable developmental or preliminary clinical information and are identified explicitly as non-peer-reviewed sources in the text.

### Data extraction and synthesis

For each primary study, basic information on model/population, target tissue, histotripsy approach and key findings were extracted. Due to substantial heterogeneity in models and limited human cases, no formal risk of bias or quality appraisal was performed. Findings are presented narratively with emphasis on consolidation of current knowledge and clinical implications for localised RCC as opposed to treatment efficacy analysis.

## Results

The structured search identified 17 in vivo renal studies forming the principal evidence base for this review. Complete database-specific search strategies, record yields, and broad reasons for exclusion are presented in Supplemental Appendix S1. The evidence is discussed according to histotripsy mechanism, renal preclinical studies, early human renal evidence, and clinical implementation considerations. An overview of the studies is provided in Table 1.

**Table 1.** Overview of in vivo renal histotripsy studies and registered clinical trials.

Series	Author	Model	Histotripsy type	Study highlights
SECTION A — Preclinical: Normal Kidney, Collecting System & Safety				
Rabbit normal kidney	Roberts et al.^24^	White rabbits normal kidney	Cavitation-cloud histotripsy	Nonthermal cavitation pulses produced sharply confined renal cavities with intact capsule, vessels and collecting system
	Hall et al.^25^	White rabbits normal kidney	Cavitation-cloud histotripsy	Follow-up histology showed cavitated renal lesions gradually resorbing and scarring with preserved global architecture
	Qi et al.^26^	White rabbits normal kidney	Two-stage millisecond-pulse histotripsy (dual-frequency)	Dual-frequency millisecond pulses achieved controllable renal lesions while maintaining nonthermal mechanisms under ultrasound guidance
Porcine ureter/pelvis safety	Kisting et al.^27^	Pigs normal proximal ureter and renal pelvis targeted	Cavitation-cloud histotripsy	Porcine survival study showed proximal ureter and pelvis histotripsy healed without strictures or obstruction
Porcine anticoagulated	Mauch et al.^28^	Anticoagulated pigs normal kidney and liver	Cavitation-cloud histotripsy	Hepatic and renal histotripsy proved feasible in anticoagulated pigs with acceptable haemodynamic stability
Swine comparative ablation	Couillard et al.^29^	Pigs normal kidney	Cavitation-cloud histotripsy vs cryoablation comparator	In swine kidneys, histotripsy matched cryoablation volumes but caused less perinephric fibrosis and distortion
Porcine BH renal/liver	Ponomarchuk et al.^30^	Pigs kidney and liver	Boiling histotripsy	Respiratory-motion work quantified targeting errors and validated gating and motion-compensation strategies for renal BH
	Song et al.^31^	Pigs kidney and liver	Boiling histotripsy	Doppler ultrasound metrics correlated with BH liquefaction volume, suggesting a real-time endpoint for treatment completeness
	Ponomarchuk et al.^32^	Ex vivo human and porcine kidneys and in vivo swine kidneys	Boiling histotripsy	BH dose studies linked exposure parameters to renal liquefaction and elastography changes, proposing imaging surrogates for dose
	Khokhlova et al.^33^	Pigs normal kidney and liver	Boiling histotripsy	BH ablated porcine liver and kidney with well-confined lesions and minimal skin effects
Porcine RAST renal ablation	Knott et al.^34^	Pigs normal kidney	Cavitation-cloud histotripsy (robotically-assisted sonic therapy)	Robotically assisted sonic therapy delivered accurate non-invasive renal ablations with automated respiratory compensation
SECTION B — Preclinical: Tumour-Bearing Models				
Rabbit orthotopic kidney	Styn et al.^35^	White rabbits orthotopic VX2 renal tumours	Cavitation-cloud histotripsy	Histology confirmed near-complete VX2 tumour fractionation with a thin inflammatory rim and limited parenchymal damage
	Styn et al.^36^	White rabbits orthotopic VX2 renal tumours	Cavitation-cloud histotripsy	Metastasis study found renal histotripsy did not increase pulmonary metastatic burden in VX2 rabbits
Eker rat RCC	Schade et al.^22^	Eker rats Tsc2-mutant hereditary RCC model	Boiling histotripsy	BH of Eker RCC triggered systemic inflammatory and CD8+ T-cell responses with precise tumour liquefaction
SECTION C — Human Evidence				
Human RCC clinical	Wah et al.^37^	Adult patient with cT1a renal cell carcinoma	Cavitation-cloud histotripsy (investigational system)	First human RCC case showed complete non-invasive ablation and preserved renal function at one-year follow-up
	Wah et al.^38,39^	Adults with non-metastatic solid renal tumours ≤3 cm	Cavitation-cloud histotripsy (investigational systems)	CAIN tests feasibility, safety and technical success of renal histotripsy for small non-metastatic tumours
	HistoSonics, Inc.^40^	Adults with non-metastatic solid renal tumours ≤3 cm	Cavitation-cloud histotripsy (Edison system)	HOPE4KIDNEY evaluates effectiveness and long-term safety of Edison renal histotripsy in a multicentre cohort

Conference abstracts are not included in this table.

Abbreviations: BH, boiling histotripsy; CAIN, Clinical Ablation Using Histotripsy for Noninvasive Treatment of Renal Tumours; RCC, renal cell carcinoma.

### Overview of histotripsy mechanism

Histotripsy, a term derived from Greek meaning ‘tissue breakdown’, uses focused ultrasound to mechanically disintegrate tissue rather than inducing thermal coagulation or freeze–thaw necrosis.^41,42^

The central mechanism is acoustic cavitation, which is the formation of small cavities such as bubbles that isolate and destroy tissue. Histotripsy systems deliver very short, high-amplitude ultrasound pulses at a low duty cycle (typically <50 microseconds), generating a high peak negative pressure at the focal point.^43^ When this negative pressure exceeds a tissue’s intrinsic cavitation threshold, it destabilises microscopic pre-existing gas nuclei that are thought to reside in tissue fluids and micro-crevices. These gas nuclei rapidly expand into a dense bubble cloud that remains confined to the focal point.^41^

Once cavitation has been achieved, bubbles undergo rapid cycles of expansion and collapse via pressure changes. This process generates localised mechanical stress such as shockwaves, high transient stresses and micro-jetting at the bubble cloud. With repeated cycles this produces mechanical fractionation through cell membrane rupture, thereby disrupting tissue architecture, and the treated volume transitions toward a relatively uniform, largely acellular homogenate.^44,45^ The ability to achieve cavitation or bubbles, is tissue dependent and based on acoustic properties. Generally, cavitation thresholds are higher in water-based tissues (around 26–30 MPa) and lower in fat-based tissues (14–17 MPa).^46^

Histotripsy can be delivered via two distinct cavitation strategies: cavitation-cloud approaches and boiling histotripsy (BH).^47^ Cavitation-cloud histotripsy generates a dense cluster of microbubbles and can be initiated either by intrinsic-threshold histotripsy or shock-scattering histotripsy. Intrinsic-threshold histotripsy uses a single pulse waveform with a large negative-pressure phase that exceeds the cavitation threshold of the target tissue.^41^ By contrast, shock-scattering histotripsy initially forms a microbubble that is then amplified into a cloud by scattered shockwaves directly reflected upon that initial bubble.^48^ Boiling histotripsy begins differently. It uses ultrasound energy to produce rapid localised heating within a small tissue region, generating a boiling bubble that expands into a vapour cavity.^49^ As the cavity enlarges, it shears adjacent tissue and expels fine tissue fragments (acoustic atomisation), progressively leading to cellular destruction and liquefaction.^50,51^ Treatment times per focal zone are also shorter with boiling histotripsy, typically between 1–30 seconds, compared with up to several minutes for conventional cavitation-cloud histotripsy. Notably, while heat is used to initiate the vapour cavity, the dominant ablative effect is mechanical and can extend well beyond the initially heated volume.

In the clinical context, intrinsic-threshold histotripsy is the preferred method as it achieves reliable cavitation while limiting unwanted heating.^52^ Control of the treatment zone is determined by pulse parameters (pressure amplitude, pulse duration, pulse repetition frequency) and tissue characteristics with typical focal zones being small (e.g., 1–2 mm by 2–4 mm).^53^ Targets are treated by “painting” the volume with repeated exposures, using mechanical or electronic steering. Real-time ultrasound can often visualise this process with the bubble cloud, providing immediate feedback, precision and reducing off-target cavitation (collecting system/segmental vessels).^21^ Post-treatment, tissue evolution following histotripsy also differs from thermal ablation. Rather than leaving a persistent solid necrotic mass, the liquefied acellular debris is gradually resorbed and remodelled over weeks to months, ultimately resulting in a smaller fibrotic scar.^54^ Supplemental Figure S1 provides a schematic overview of the histotripsy procedure, including patient positioning, transcutaneous ultrasound delivery and image-guided focal cavitation.

### Pre-clinical studies

In vivo renal histotripsy literature generally divides into normal-kidney experiments that established basic feasibility, safety and dosimetry, and tumour-bearing models that began to address oncologic efficacy and immunology.

Early work began in small animal models using cavitation-cloud histotripsy in normal rabbit kidneys. Roberts et al. first showed that pulsed cavitational ultrasound could create sharply demarcated, non-thermal cavities in the renal parenchyma with a liquefied core and a narrow rim of cellular injury. This established the feasibility of purely mechanical ablation in renal tissue.^24^ Hall and colleagues then mapped temporal histology evolution, demonstrating predictable progression from acute acellular debris to granulation tissue and fibrosis without major off-target injury, suggesting that kidneys can remodel after focal mechanical liquefaction.^25^

Once basic parenchymal behaviour was understood, work moved to orthotopic tumour models. Styn and colleagues first reported feasibility of ablating VX-2 tumours implanted in rabbit kidneys, achieving fractionation in most treated tumours (16/20). Examination of histologic effects and perilesional changes showed sharply confined ablation cavities with only a thin rim of haemorrhage and inflammation.^35^ In a follow-up metastasis study, mechanical tumour disruption was not associated with a statistically significant increase in lung metastasis burden compared to controls. Together, the VX-2 data suggested that renal histotripsy can debulk solid tumours without significant evidence of promoting metastasis.^36^

The Eker rat series introduced boiling histotripsy and established that BH can liquefy spontaneous RCCs in Tsc2-mutant models, producing sharply demarcated cavities that include both tumour and a narrow rim of adjacent parenchyma.^22^ The same study documented acute immunological changes after treatment, with transient elevations in the damage-associated molecular pattern high mobility group box 1 (HMGB1) and tumour necrosis factor (TNF), and increased CD8+ T-cell infiltration in both treated and contralateral tumours, suggestive of a systemic immune response.^22^ A chronic-model report, available only in abstract form, further suggested durable tumour debulking without apparent acceleration of residual growth after partial treatment, though both the durability of debulking and the immune signal remain to be confirmed beyond these early models.^55^

In parallel, studies moved into large-animal porcine models to address transcutaneous delivery and human-scale organ size. Khokhlova and colleagues led a sequence of porcine works exploring volumetric BH ablation of liver and kidney, demonstrating precise cavities through intact abdominal wall and preserved overlying skin and ribs. Pilot porcine BH kidney ablations using clinical-style systems also described reproducible cavities with limited perinephric haematoma.^33^ Complementing these studies, Knott et al. introduced a robotically assisted sonic therapy (RAST) system based on histotripsy that automated three-dimensional renal ablations.^34^ Together, these studies showed that histotripsy could be delivered reproducibly on clinical hardware with sophisticated guidance.

More recently, studies refined pulse design and assessed “edge-case” safety. Qi et al. developed millisecond two-stage pulses in rabbit kidneys, combining fundamental and second harmonic components, and demonstrated ellipsoidal lesions with an approximately seven-fold higher ablation rate versus single-frequency pulses, potentially improving treatment efficacy.^26^ Ponomarchuk et al. then quantified a “BH dose” via dose-escalation and shear-wave elastography, demonstrating that perfused kidneys (in vivo) require lower doses to achieve comparable fractionation endpoints.^32^ A companion study quantified the impact of respiratory motion and evaluated gating or motion-mitigation strategies as efficacious in improving fractionation completeness.^30^ Song et al. proposed Doppler-based monitoring of bubble cloud dynamics, by correlating Doppler colour signatures with volumetric liquefaction and potentially serving as feedback on treatment success.^31^

Safety around vessels, coagulation and the collecting system has been addressed systematically. Mauch et al. evaluated hepatic and renal histotripsy in warfarin-anticoagulated pigs and produced well-confined cavities with transient haematuria and without significant haemorrhage, suggesting clinically relevant anticoagulation may not preclude safe histotripsy.^28^ Kisting et al.’s survival model targeting the proximal ureter and renal pelvis further reassured on central collecting-system safety, with histotripsy causing only transient urothelial injury with complete histologic recovery and no long-term loss of renal function.^27^ In a direct comparison with cryoablation, Couillard and colleagues achieved equivalent ablation volumes, markedly fewer perirenal haematomas, and similar serum creatinine changes by day 30. Overall, these findings suggest a similar safety profile in chronic swine models.^29^

### Renal clinical studies

Building on this promising preclinical foundation, early-phase human studies are ongoing and moving beyond pure feasibility. Clinical Ablation Using Histotripsy for Noninvasive Treatment of Renal Tumors (CAIN, NCT05432232) is the first trial and to date, provides the most detailed dataset.^38^ CAIN is a prospective, multi-centre, single-arm pilot study of the HistoSonics investigational system in adults with non-metastatic solid renal tumours ≤3 cm and preserved renal function, defined as an estimated glomerular filtration rate (eGFR) of at least 45 mL/min. Primary endpoints are (i) technical success: complete tumour coverage on computed tomography (CT) or magnetic resonance imaging (MRI) within 36 h and (ii) freedom from Clavien–Dindo ≥III complications at 30 days. Patients undergo follow-up imaging to 180 days, with the aims being to assess safety, technical success, and preliminary efficacy.

Preliminary first-in-human findings from the CAIN feasibility study have been reported in conference form and should therefore be interpreted as non-peer-reviewed data.^56^ Of 20 screened patients, 11 underwent treatment. Median tumour size was ∼2.4 cm and lesions were mainly low-complexity by RENAL nephrometry score, and interpolar or lower-pole masses. Technical success at 36 hours was achieved in all 11 treated patients and was defined in the published protocol as complete planned tumour coverage on contrast-enhanced CT or MRI.^39^ A 30-day efficacy of 90% was also presented, although further detail regarding the evaluable denominator and assessment criteria was not specified. Major complications were uncommon, with one grade IIIa event related to prolonged head-down positioning rather than the device; other minor events were largely transient haematuria. Practical constraints were also highlighted, such as general anaesthesia, treatment duration approaching 80 minutes, and the need for careful respiratory management and acoustic window optimisation.^37^ These preliminary findings support procedural feasibility in carefully selected small tumours but do not establish comparative safety, oncological efficacy, or applicability to complex renal masses.^56^

Complementing the preliminary trial-level data, Wah et al. have also published a one-year case report of a CAIN participant. A 2.8 cm lower-pole, low-complexity RCC was treated with histotripsy over 83 minutes; 36-hour CT confirmed complete non-enhancing ablation, and one-year imaging demonstrated no residual or recurrent disease with progressive involution of the ablation cavity. Complications experienced were grade I events and self-limiting (haematuria, lower abdominal pain and nausea). Although anecdotal, this case offers the first glimpse of longer-term local control and parenchymal remodelling in human kidney tissue.^37^

A second trial, The HistoSonics Edison System for Treatment of Primary Solid Renal Tumors Using Histotripsy (#HOPE4KIDNEY, NCT05820087) extends this programme into a larger, multicentre, single-arm study of the Edison™ system.^40^ It is designed to assess efficacy and safety of non-invasive destruction of kidney tissue and aims to summarise 90-day outcomes for all enrolled patients, with continued surveillance out to five years. Recruitment has now closed, but results have yet to be reported publicly. The study commenced in January 2024, with primary completion in September 2025 and secondary completion expected by July 2030.

### Non-renal clinical studies

Clinical use of histotripsy has been explored for various benign and malignant indications, almost exclusively in early-phase, single-arm studies. Collectively, these trials serve to provide more information regarding procedural safety, technical performance and device regulation as opposed to comparative oncologic benefit.^57^

The earliest indication was for benign prostatic hyperplasia, where 25 men were treated using the Vortx Rx system.^58^ While lower urinary tract symptoms improved (mean International Prostate Symptom Score (IPSS) reduction of approximately 10 points at 6 months), there was no clear endoscopic or ultrasound evidence of gland debulking, no meaningful change in uroflow or post-void residual, and four patients developed urinary retention. Importantly however, no serious intraoperative complications occurred, thereby establishing procedural safety of cavitation-based therapy in the urologic setting, despite modest clinical efficacy.^58^

Oncologic applications have largely focused on liver tumours.^59^ The THERESA phase I trial in Barcelona (8 patients, 11 lesions) demonstrated that histotripsy could reproducibly create non-enhancing ablation zones matching the planned target on MRI 24 hours post procedure, with no device-related complications. Two instances of local progression were attributed to incomplete coverage or competing adjacent growth, highlighting the importance of image guidance and targeting on treatment efficacy as opposed to energy delivery itself.^60^

Subsequently, the larger #HOPE4LIVER programme, which pooled the #HOPE4LIVEREU/UK and #HOPE4LIVERUS trials, reported outcomes leading to US Food and Drug Administration (FDA) approval of the Edison system.^61^ In the first 44 patients, tumour-level technical success was 95%, with major complications in 7%, all consistent with known risks of liver ablation. A subsequent multicentre safety analysis of 230 post-approval cases reported complications in 5%, with major events attributed to disease progression rather than treatment-related injury. A one-year update from #HOPE4LIVER showed local control and survival broadly comparable to other liver-directed therapies in similar-stage hepatocellular carcinoma (HCC) and metastatic disease.^62,63^

## Discussion

### Mechanistic Advantages

Percutaneous ablation offers a minimally invasive alternative for RCC patients in whom surgery carries prohibitive risk.^10^ Within this landscape, histotripsy is a promising modality clinically distinct from established ablation methods. Its effect is predominantly mechanical, producing a sharply bounded region of liquefaction with a small transition rim, rather than a broad thermal penumbra with a wide transition zone of partially damaged tissue.^59^ Histotripsy cavitation occurs only once focal peak negative pressure exceeds a tissue-specific threshold, and collagen-rich structures (vessel walls and the renal pelvis) have a higher threshold for disruption than tumour. As such, histotripsy reduces collateral injury and may be particularly advantageous in nephron-sparing applications where the collecting system, sinus fat and vessels lie proximal to tumour.^41^ Histotripsy also appears less affected by heat-sink effects, with both pre-clinical and early clinical experience suggesting ablation may be performed adjacent to vessels without the predictable loss of margin seen with thermal techniques.^64^ In addition, as the delivery is external, it avoids tract-related complications of percutaneous access with the added benefit of real-time ultrasound guidance.^37,56^

### Comparative context and evidence maturity

Established focal therapies have a substantially more mature evidence base than renal histotripsy. In a 2025 systematic review and meta-analysis of 133 studies including 8,910 patients, five-year local-control estimates for tumours smaller than 4 cm were 95% for RFA, 92% for MWA, 91% for cryoablation, and 98% for SBRT. Grade 3–4 adverse-event rates across the overall pooled cohorts were 2% for RFA, 1% for MWA, 3% for cryoablation, and 2% for SBRT. These estimates offer a benchmark against which the small tumours enrolled in CAIN can be considered, though they derive predominantly from non-randomised cohorts with heterogeneous baseline characteristics, outcome definitions, and follow-up, and should not be read as direct comparisons of modality superiority.^10^

The CAIN cohort represents the favourable end of this spectrum, comprising predominantly low-complexity interpolar or lower-pole lesions.^56^ Early imaging-defined technical success is encouraging, but cannot establish superiority, or even equivalence, to modalities supported by multi-year local-control and safety data. Whether the mechanical, non-thermal ablation and its relative heat-sink insensitivity of histotripsy translate into improved margins or local control for perivascular, hilar, or central renal tumours remains unproven in comparative clinical studies.^17,18,21,37^ Beyond local control, direct comparative evidence between histotripsy and established focal therapies also remains absent across treatment delivery and anaesthetic requirements, renal functional and patient-reported outcomes, and resource use and cost-effectiveness.

Histotripsy should not be assessed solely against other interventional modalities. Active surveillance remains a relevant comparator for selected patients with cT1a tumours; prospective multi-institutional Delayed Intervention and Surveillance for Small Renal Masses (DISSRM) data showed no significant difference in cancer-specific survival between surveillance and primary intervention.^4,8,65^ Any future role for histotripsy would therefore likely be confined to patients with an indication or preference for active treatment in whom surgery is unsuitable or declined, with surveillance forming part of counselling alongside surgery and established focal therapies.

### Practical and clinical challenges

The role of histotripsy is also shaped by several practical constraints, the most significant being acoustic access and target motion. Ultrasound requires usable windows - gas interfaces (bowel, lung) and bone (ribs and spine) restrict energy delivery. Rib shadowing, overlying bowel gas, and body habitus can narrow the effective aperture and limit targeting options.^21^ Even with an appropriate window, motion, often from respiration, can smear the focal zone and create partially treated regions unless gating or motion-tracking strategies are used.^30^ Current mitigating solutions such as jet ventilation and motion-compensation algorithms remain technically demanding and are not yet standardised.^66^

Tissue heterogeneity also complicates dose planning. The renal cortex, medulla and sinus fat differ in acoustic properties and cavitation thresholds, potentially contributing to variability in lesion fractionation, especially for larger volumes.^29,32^ Proposed refinements, including dual-frequency pulse schemes and elastography-derived response metrics are promising but remain investigational.^26^

While histotripsy has been shown to be relatively low risk, portal and hepatic vein thrombi have been observed in liver studies. These events have been attributed to cavitation-induced platelet activation and have resolved within a 4-week follow-up period. However, long-term information on vascular implications and whether these findings extrapolate to renal venous anatomy remains limited.^67^

Like other non-resective focal therapies, histotripsy does not yield a post-treatment specimen or pathological margin assessment. Histotripsy is not specifically addressed in current guidelines; however, the CAIN protocol required renal mass biopsy at least 14 days before treatment, consistent with recommendations for established thermal ablation.^4,11^ Treatment response is therefore assessed radiologically: CAIN defined technical success as complete planned tumour coverage on contrast-enhanced CT or MRI within 36 hours, whereas technique efficacy was assessed at 90 days.^39^ These early imaging endpoints are distinct from durable local control, which requires longer-term surveillance for residual or recurrent enhancing disease. Histotripsy-specific surveillance and salvage pathways remain undefined, and management of suspected residual or recurrent disease would likely be extrapolated from established renal-mass care.^4,8^

Overall, the evidence base remains early but growing. Most published clinical data to date come from single-arm, early-phase studies that prioritise feasibility, technical success and short-term safety, rather than long-term oncologic endpoints.^56,61,63^ As a result, long-term local control, metastasis-free survival, and renal function trajectories are not yet well characterised. Clinical workflows are also still being refined, with many protocols relying on general anaesthesia, detailed planning and relatively long treatment times.^37,39^ Comparative effectiveness against partial nephrectomy and ablative techniques has yet to be addressed and is increasingly positioned as the next step as multi-centre experience and prospective datasets mature.^57,68^

### Limitations

This review should be interpreted in the context of its design and the available evidence base. As a narrative synthesis informed by a structured search, it did not involve duplicate independent screening, formal risk-of-bias assessment, or quantitative pooling. The renal evidence base is small and heterogeneous, with human experience confined to a single peer-reviewed case report and a conference-reported feasibility cohort that has not yet been peer reviewed in full. Much of the preclinical evidence also derives from animal and normal-tissue models of uncertain relevance to human renal cell carcinoma. Accordingly, this review is intended to contextualise the current translational evidence and its clinical uncertainties, rather than to determine comparative effectiveness or define a treatment algorithm for renal tumours.

## Conclusion

Histotripsy for renal malignancy has progressed from proof of concept in preclinical animal models to clinical use in selected tumours. It offers a promising non-invasive modality that addresses several limitations of surgical and ablative treatments for suitable RCC. Taken together, animal data and early phase human trials demonstrate histotripsy can achieve complete coverage of small renal lesions with acceptable peri-procedural risk and preservation of renal function. Ongoing trials and future studies are essential to capture long-term oncologic and functional outcomes, extend evaluation to more complex tumours, and to explore potential synergies with systemic and immunotherapies. If HOPE4KIDNEY results confirm preliminary feasibility and safety, histotripsy may become an additional option within the focal therapy armamentarium for selected patients with small renal masses.

## Supplemental material

Supplemental material - Histotripsy for Renal Tumours: A narrative review of preclinical and early clinical evidence Supplemental material for Histotripsy for Renal Tumours: A narrative review of preclinical and early clinical evidence by Anirudh Singh Bhati, Ankur Patel, TAN Ru Yu TAN, Valerie Gan, Apoorva Gogna in Therapeutic Advances in Urology.

Supplemental material - Histotripsy for Renal Tumours: A narrative review of preclinical and early clinical evidence Supplemental material for Histotripsy for Renal Tumours: A narrative review of preclinical and early clinical evidence by Anirudh Singh Bhati, Ankur Patel, TAN Ru Yu TAN, Valerie Gan, Apoorva Gogna in Therapeutic Advances in Urology.

## Data Availability

Data sharing not applicable to this article as no datasets were generated or analysed during the current study. Search strategies generated are available as supplementary information files.*
