# Supplementary material for: Histotripsy for Renal Tumours: A narrative review of preclinical and early clinical evidence
Source: Ther Adv Urol. 2026 Oct 7;18:17562872261485822. doi: 10.1177/17562872261485822 (PMC13769914; doi:10.1177/17562872261485822)
Supplement: Supplementary file 1 [file sj-pdf-1-tau-10.1177_17562872261485822.pdf]

Supplementary Appendix S1

Full electronic search strategy

**Databases searched**	PubMed, Scopus, and Embase
**Search date**	14 December 2025

Embase

**Database**	Embase Classic+Embase via Ovid
**Final search**	101

#	Query	Results from 14 Dec 2025
1	histotripsy.ti,ab.	643
2	"boiling histotripsy".ti,ab.	98
3	(histotripsy or "boiling histotripsy" or "cavitation cloud" or "mechanical hifu" or "mechanical high intensity focused ultrasound" or "non-thermal hifu").ti,ab.	706
4	exp kidney/ or exp kidney tumor/ or exp renal cortex/ or exp renal medulla/ or exp renal pelvis/ or exp ureter/ or (kidney or kidneys or renal or nephron* or "renal pelvis" or ureter*).ti,ab.	1,993,683

#	Query	Results from 14 Dec 2025
5	3 and 4	101

PubMed

**Final search**	60

**Search strategy**
(histotripsy[Title/Abstract] OR "boiling histotripsy"[Title/Abstract] OR "cavitation cloud"[Title/Abstract] OR "mechanical HIFU"[Title/Abstract] OR "mechanical high intensity focused ultrasound"[Title/Abstract] OR "non-thermal HIFU"[Title/Abstract]) AND ("Kidney"[MeSH Terms] OR kidney[Title/Abstract] OR renal[Title/Abstract] OR nephron[Title/Abstract] OR "renal pelvis"[Title/Abstract] OR ureter*[Title/Abstract])

Scopus

**Final search**	64

**Search strategy**
TITLE-ABS-KEY (histotrips* OR "boiling histotripsy") AND TITLE-ABS-KEY (kidney OR kidneys OR renal OR nephro* OR "renal pelvis" OR ureter* OR "renal cell carcinoma" OR RCC) AND DOCTYPE (ar OR ip OR cp)

Study selection and record yields

The study selection process is summarised in Supplementary Figure S2. The search returned 225 records, and 101 remained after 124 duplicates were removed. Screening of titles and abstracts excluded 76 records as outside the scope of this review: those that did not evaluate histotripsy, targeted non-renal tissue, were limited to ex vivo or phantom models, applied thermal high-intensity focused ultrasound without a mechanical histotripsy regimen, or constituted non-primary literature such as reviews and commentaries. Of the remaining 25 articles assessed in full, 8 were excluded because the histotripsy target was non-renal or not examined in vivo, leaving 17 studies that form the evidence base for this review.

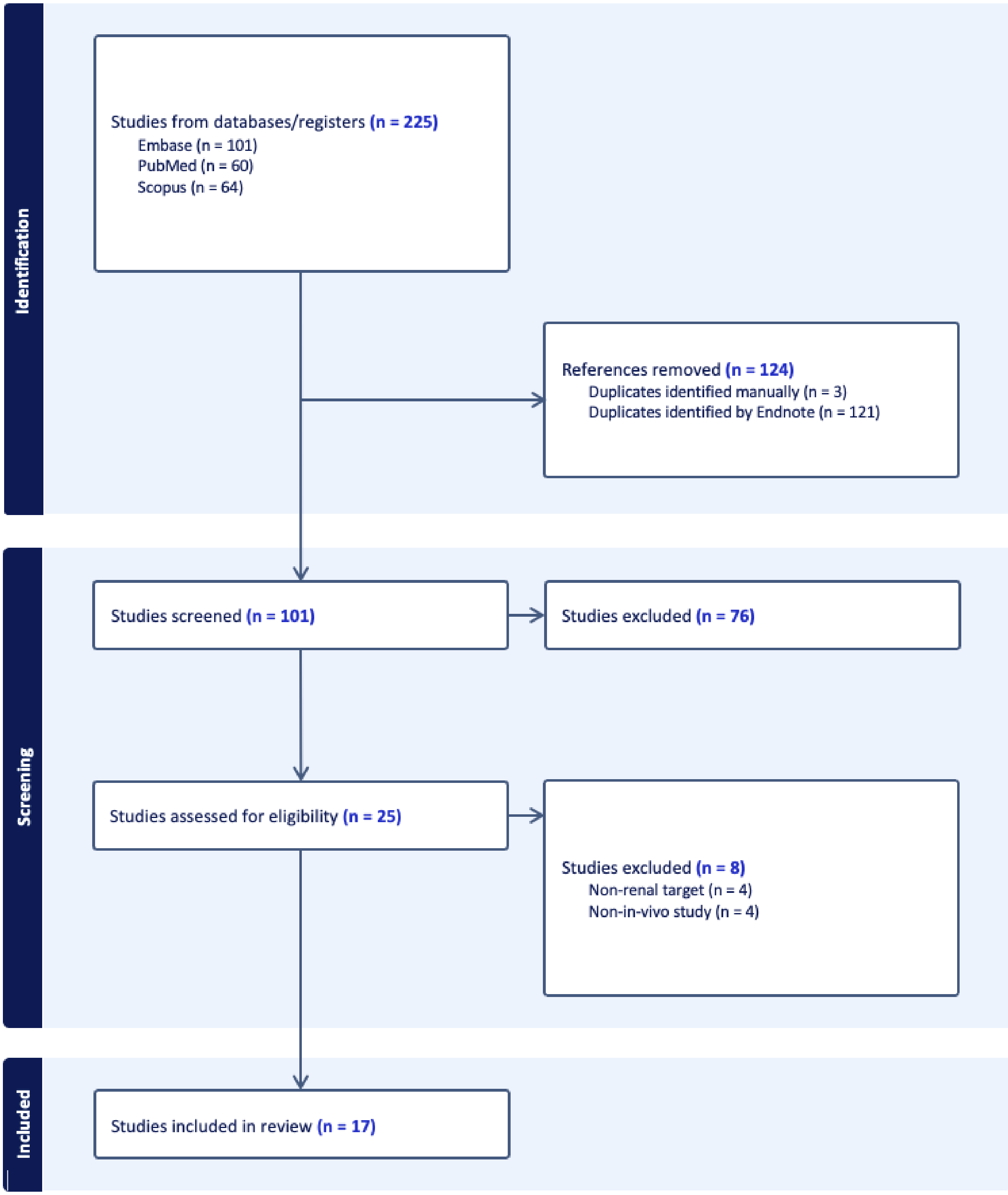

Supplementary Figure S2: Flowchart showing study identification and selection
