# Supplementary material for: Histotripsy for Renal Tumours: A narrative review of preclinical and early clinical evidence
Source: Ther Adv Urol. 2026 Oct 7;18:17562872261485822. doi: 10.1177/17562872261485822 (PMC13769914; doi:10.1177/17562872261485822)
Supplement: Supplementary file 2 [file sj-pdf-2-tau-10.1177_17562872261485822.pdf]

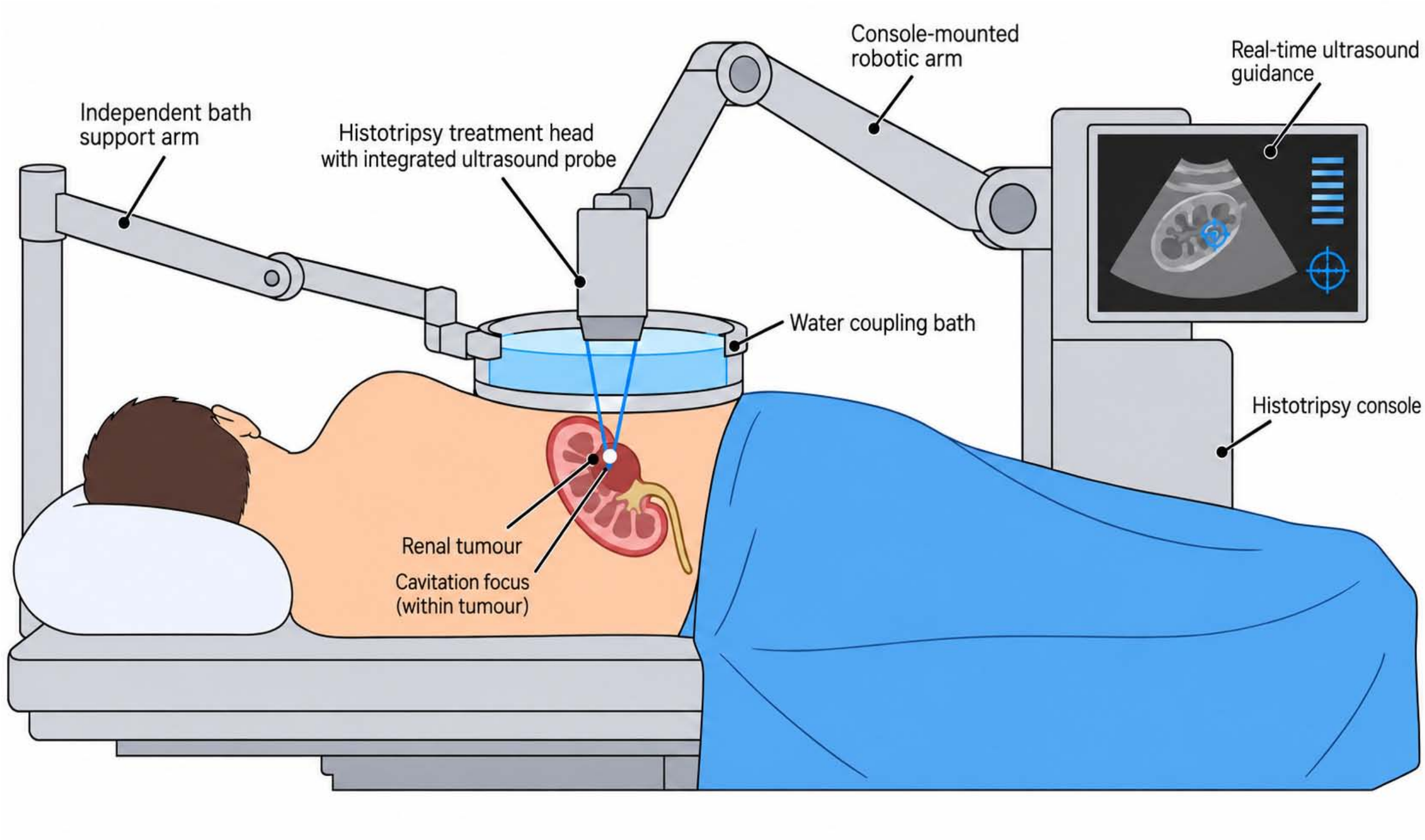

Supplementary Figure S1: Simplified schematic of renal histotripsy. The patient is positioned to permit an acoustic window to the renal tumour. A transcutaneous focused ultrasound source delivers short, high-amplitude pulses to the target under image guidance, generating confined cavitation at the focus that fractionates tissue within the planned treatment volume. The schematic is illustrative and not intended to represent a specific commercial system or procedural protocol.
